# Recent Advances in Assessment and Rehabilitation of Individuals with Communication and Language Disorders

**DOI:** 10.3390/brainsci15101095

**Published:** 2025-10-11

**Authors:** Dionysios Tafiadis, Angelos Papadopoulos, Louiza Voniati, Nafsika Ziavra

**Affiliations:** 1 Department of Speech & Language Therapy, School of Health Sciences, University of Ioannina, 45100 Ioannina, Greece; angelospapadopoulos@gmail.com (A.P.); nziavra@uoi.gr (N.Z.); 2 Department of Health Sciences, Speech and Language Therapy, European University, 1516 Nicosia, Cyprus; l.voniati@euc.ac.cy

## 1. Introduction

Currently, in the field of rehabilitation, there is a need for researchers and clinicians to stay updated on recent knowledge worldwide. Often, this is crucial for planning a treatment for individuals with communication and language disorders. Therefore, many researchers recognize the need for an update to the existing literature for various disorders, such as aphasia, autism spectrum disorders, and genetic syndromes [1,2,3,4,5]. Moreover, it is also beneficial for professionals, and even more so for academics, to dedicate their best and most up-to-date efforts to understanding how individuals develop, use, and recover language throughout the lifespan.

The current Editorial refers to the Special Issue “Recent Advances in Assessment and Rehabilitation of Individuals with Communication and Language Disorders”. The Special Issue highlights new opportunities and challenges for advancing assessment and rehabilitation for individuals with language and communication disorders, with a specific focus on interdisciplinary research related to neurodevelopmental disorders, neurodegenerative disorders, and other related conditions. As indicated in recent years, researchers have increased the emphasis that various factors (cognitive, neurological, and environmental) have an impact on neurodevelopmental disorders, neurodegenerative disorders, and other related disorders.

The contributors of this Special Issue featured various studies (reviews, systematic reviews, and original articles). Nineteen (19) manuscripts were submitted for consideration for the Special Issue. Specifically, five (5) original articles, six (6) reviews, one (1) longitudinal case report, and one (1) brief report. One contributor study was selected as the editor’s choice. The contributions are listed below.

Regarding the contributions in original articles, Sanhueza-Garrido et al. Adapted the Bogenhausen Dysarthria Scales (BoDyS) to Chilean Spanish speakers. In Greece, Zarokanellou et al. explored the narrative ability in Greek-speaking children with High-functioning autism spectrum disorders. Agbavor and Liang conducted research about multilingual prediction of cognitive impairment with large language models and speech analysis. Bachourou et al. studied cognitive vs. linguistic training in children with developmental language disorder by exploring their effectiveness on verbal short-term memory and verbal working memory. Trimmis et al. focused on creating a Greek Pediatric Word Recognition Test (GPWR) by picture identification.

Furthermore, there were six (6) reviews, one (1) brief report, and one (1) longitudinal case report. Voniati et al. published their narrative review on communication abilities, assessment procedures, and intervention approaches in Rett Syndrome. Papadopoulos et al. conducted a scoping review about the effectiveness of Lee Silverman Voice Treatment (LSVT LOUD) on children’s speech and voice. Voity et al. proceeded with an update on how to approach a patient with Locked-In Syndrome and their communication ability. Ericson et al. conducted a systematic review of computer- and smart-tablet-based self-administered treatments in chronic post-stroke Aphasia. A systematic review was published by Georgopoulou et al. about the efficacy of rTMS combined with cognitive and language training in people living with Alzheimer’s Disease. Another systematic review by Vlachou et al. about the evidence of language development using brief animated stimuli. Moreover, a Brief Report was published by Yoo on the dynamics of inferential behavior while reading expository and narrative texts. Finally, a longitudinal case report of semantic variant of primary progressive Aphasia (svPPA) was published by Strunk, Weiss, and Müller, focused on high-frequency language therapy with semantic feature analysis (SFA) and transcranial direct current stimulation (tDCS).

## 2. An Overview of Published Articles

The first contributor study aimed to cross-culturally adapt the Bogenhausen Dysarthria Scales (BoDyS) into Chilean Spanish and to conduct face and content validation. The adaptation process resulted in a consensus version that retained the semantic and cultural characteristics of the original scale. Both the face and content validity were evaluated by a panel of experts consisting of ten Chilean speech–language pathologists (eight women and two men), who were selected through convenience sampling. For the pilot study, 10 new experts were selected through convenience sampling, all working in different healthcare centers in Chile. Moreover, the statistical measures indicated satisfactory levels of agreement, and the pilot study demonstrated the scale’s appropriateness and effectiveness for assessing dysarthria within the Chilean context. However, some experts recommended reducing task repetition for patients prone to fatigue. The Chilean version of the BoDyS seems to be a valid and valuable tool for evaluating dysarthria in Chile.

The second contributor study investigated the narrative ability of monolingual Greek-speaking HF-ASD children in comparison to that of their typically developing (TD) peers. Specifically, it explored the associations between narrative variables, ADHD symptomatology, and memory skills in the participants on the autistic spectrum. In the study, 39 children aged 7 to 12 years participated, 19 with HF-ASD and 20 age-matched, vocabulary-matched, and cognitively matched TD peers. The two groups were similar in most microstructural and macrostructural variables. Still, they differed significantly in syntactic complexity and subordination indices, indicating that the HF-ASD group exhibited syntactic delay compared to their TD peers. The HF-ASD participants showed statistically significantly higher heterogeneity in the amount of information generated for the story’s main character in comparison to their TD peers. Moreover, significant associations were observed between verbal and visual memory, complex syntactic structures, and terms related to the Theory of Mind and internal state. ADHD symptomatology was negatively correlated with the generation of coordinated and straightforward clauses. Finally, complex syntax and delayed verbal short-term memory (vSTM) were correlated with retelling total scores, indicating that language ability and verbal memory compensate for narrative competence in HF-ASD children. The study’s findings highlight the impact of language skills, memory ability, and ADHD symptomatology on narrative competence in children with HF-ASD, as well as the importance of narrative use in assessing language skills in populations with mild language impairment.

The third contributor study presented results from the INTERSPEECH TAUKADIAL Challenge data set, where the aim was to automatically detect mild cognitive impairment (MCI) and predict cognitive scores for English and Chinese speakers. Their approach leverages Whisper, a speech foundation model, to extract language-agnostic speech embeddings. Then, utilize ensemble models to incorporate task-specific information. The model achieved an unweighted average recall of 81.83% in an MCI classification task and a root mean squared error of 1.196 in the cognitive score prediction task, which placed the model in second and first position, respectively, in the ranking for each task. A comparison between language-agnostic and language-specific models reveals the importance of capturing language-specific nuances for the accurate prediction of cognitive impairment. This study demonstrates the effectiveness of language-specific ensemble modeling with Whisper embeddings in enabling scalable, non-invasive cognitive health assessments of Alzheimer’s disease, achieving state-of-the-art results in multilingual settings.

The fourth contributor study explored comparatively the effectiveness of a cognitive (verbal short-term memory (vSTM), verbal working memory (vWM)) and of a linguistic training (10-week duration each) in the diffusion of gains in cognitive abilities (vSTM and vWM) of in-school-aged Greek-speaking children with developmental language disorder (DLD). Specifically, the researchers utilized two computerized training programs, i.e., a linguistic and a cognitive one, which were developed and applied to three groups (A, B, and C) of children with DLD (*n* = 49, in total). There were three assessments with two vSTM tasks (non-word repetition and forward digit span) and a vWM task (backward digit span): pre-therapeutically (time 1), where no significant between-group differences were found, post-therapeutically I (time 2), and post-therapeutically II (time 3), and two training phases. In phase Ι, group A received meta-syntactic training, whereas group B received vSTM/vWM training, and group C received no training. In Phase II, a reversal of treatment was performed for groups A and B: group A received vSTM/vWM, while group B received metasyntactic training. Again, group C received no training. Overall, the results indicated a significant performance improvement for the treatment groups and revealed beneficial far-transfer effects, as language therapy can affect vSTM and vWM in addition to direct and near-transfer effects. In addition, the intervention type order affected performance as follows: first, better performance on the vSTM task (non-word repetition) was shown when the linguistic treatment was delivered first; second, better performance on the vWM in Time 2 and Time 3 was shown by group B, for which the cognitive treatment was delivered first. The researchers concluded that not only the type of intervention but also the order in which it is administered can affect performance in DLD.

The fifth contributor study aimed to construct a clinically valuable closet-set WRS test with a picture identification task for young Greek-speaking children. The test material was meticulously designed in accordance with specific criteria. Then, to determine which parts of speech are used more frequently by preschool children, a spontaneous speech sample (250 words per child) was collected from 300 children aged 3 to 6 years. The researchers developed and applied two phonemically balanced 50-word lists suitable for young children, as well as created picture representations for each response set. Furthermore, all testing was conducted in an audiometric booth that exceeded the ambient noise level standards of the audiometric rooms. The speech signal was routed from a laptop computer to a GSI 61 audiometer, and all test items were delivered from the audiometer to the subject. Moreover, the results indicated that materials for a WRS test for young children are developed with high face validity and are applicable for children as young as three years old. The test satisfies the essential components needed for a WRS test. It consists of two phonemically balanced 50-word lists with low-redundancy bisyllabic words, with each list containing 227 phonemes. This closed-set WRS test presents a new and valuable tool for assessing speech perception skills in young Greek-speaking children.

The sixth contributor study aimed to synthesize the research from the last two decades regarding the verbal and nonverbal communication abilities, assessment procedures, and intervention approaches for individuals with RTT. The authors conducted a structured literature search using the Embase, Scopus, and PubMed databases, and Fifty-seven studies were selected and analyzed based on inclusion criteria. The data were categorized into four domains (verbal communication skills, nonverbal communication skills, assessment procedures, and intervention approaches). The study’s findings revealed a diverse range of communicative behaviors across the RTT population, including prelinguistic signals, regression in verbal output, and preserved nonverbal communicative intent. Moreover, the results highlighted the importance of tailored assessments (Inventory of Potential Communicative Acts, eye tracking tools, and Augmentative and Alternative Communication) to facilitate functional communication. The individualized intervention approaches were found to be the most effective in improving communicative participation. This literature review provides an overview of the last two decades of evidence with an emphasis on the need for personalized and evidence-based clinical practices to improve the quality of life for individuals with RTT.

The seventh contributor study is a scoping review with a primary goal of reviewing the literature and analyzing the possible effectiveness of the LSVT LOUD approach in children with voice and speech deficits. The authors searched the Scopus and PubMed databases in May 2024, and eleven articles were obtained from the search. The standards of the PRISMA recommendations were used for scoping reviews, and the PCC framework was employed for establishing the eligibility criteria. Furthermore, the study employed the instructions outlined in the Cochrane Handbook for quality assessment. Regarding the findings, the reviewed studies employed both formal and informal measures to assess voice and speech abilities in children, with the majority of studies including children with Cerebral Palsy (CP) and those with Down Syndrome (DS). Furthermore, all the studies reported that children with CP and DS underwent a total dose of the LSVT LOUD treatment, with significant post-treatment findings indicating increased speech function and sound pressure level, as reflected in the auditory–perceptual ratings of voice and speech improvement. In many studies, parents’ and expert listeners’ ratings of voice, perception of vocal loudness, speech, and communication indicated improvement. In conclusion, most of the studies included provide positive evidence for the LSVT as an approach. However, the small sample size that featured in the studies, as well as their limitations, made these conclusions uncertain.

The eighth contributor study focused on the Locked-in syndrome (LIS), which is a rare and challenging condition that results in tetraplegia and cranial nerve paralysis while maintaining consciousness and variable cognitive function. This article aimed to increase knowledge of how to enhance and manage communication among patients in the LIS setting. After the acute management is completed, it is essential to collaborate with the patient in developing a plan to maintain and enhance their quality of life (QOL). A key component in increasing or maintaining QOL within this population involves establishing a functional communication system. Evaluating cognition in patients with LIS is vital for assessing patients’ communication needs along with physical rehabilitation to maximize their QOL. Over the past decade or so, there has been an increase in research surrounding brain–computer interfaces aimed at improving communication abilities for patients with paralysis. This article provides an update on the available technology and protocols for finding the most effective way for patients with this condition to communicate.

In the ninth contributor study, this systematic review aimed to identify computer- and smart-tablet-based self-administered treatments and analyze the proposed interventions in terms of treatment targets, effectiveness (considering specificity, generalization, transfer, and maintenance), and clinician involvement (during and/or prior self-administered therapies). The authors used terms encompassing three main concepts (rehabilitation, self-administration, and aphasia) to search three electronic databases (Scopus, PubMed, and PsycINFO). This systematic review was conducted in accordance with the guidelines proposed by the Preferred Reporting Items for Systematic Reviews and Meta-Analyses using PRISMA guidelines2020. Regarding the process, two reviewers independently screened titles and abstracts against eligibility criteria, and three reviewers completed data extraction of the included studies. After the whole process was completed, thirty-nine studies were included in this study. In terms of treatment targets, anomia is the most treated symptom in published studies (*n* = 24). Still, the existence of promising studies for other disorders means that the targets can be broadened. Therapies are effective for trained items, and gains are maintained. There is some evidence of transfer effects for treatments targeting the sentence level. Most studies offer training sessions, previous self-administered therapy, and/or observation and monitoring sessions during therapy; more rarely, self-administered therapy is supplemented with face-to-face therapy. This systematic review is the first to focus specifically on self-administered technology-based therapies via computer or smart tablet.

The tenth contributor study focused on repetitive transcranial magnetic stimulation (rTMS) in patients with Alzheimer’s disease (pwAD), similar to multidomain cognitive training (CT). The current review aims to evaluate the cognitive benefits of combining rTMS with CT, including language training, for individuals with mild to moderate AD. An extensive literature search was conducted in PubMed, Google Scholar, and the Cochrane Library using relevant terms, resulting in nine studies with a total of 290 participants (190 in the Active Group and 100 in the Control Group). The comprehensive review of the articles revealed that the combined treatment improved global cognitive function, as well as neurocognitive, neuropsychiatric, and quality of life in the AG. Nevertheless, these results should be interpreted cautiously, given the relatively small number of existing studies on this specific combination.

The eleventh contributor study is a brief report that aimed to examine changes in inferential behavior while reading different genres. The inferential behavior of 28 students with reading disabilities (RDs) and 44 students without RDs was quantified while they read expository and narrative texts. First, the average rates of inference attempts and correct inferences were measured during reading. Then, the same rates were measured separately during early and late reading to see if there was a change in inferential behavior. The results indicated that the change in inferential behavior is dependent on the genre. While reading the expository text, both groups showed no significant change in their ability to make inferences. In contrast, while reading the narrative text, both groups showed higher rates of inference attempts, and only the students without RD showed a significant increase in correct inferences.

The twelfth contributor study aimed at systematically reviewing the available literature for evidence concerning the effect of brief animation on spoken language responses (receptive—listening or expressive—speaking) in typically developing children aged 3 to 9 years. The authors searched five databases, resulting in seven studies that were included. The characteristics of animated stimuli, the manner of presentation, and the language-related tasks were recorded, and questions were posed about the effect of brief animation on children’s receptive and expressive language abilities. This evidence suggested that animation may have a positive effect on the expressive language abilities of children compared to static pictures. Regarding the impact of animation on receptive language performance, the evidence is less conclusive.

The thirteenth study is a case report that aimed to investigate whether the combination of semantic feature analysis (SFA) and transcranial direct current stimulation (tDCS) is effective in treating word retrieval in the semantic variant of primary progressive aphasia (svPPA) and to determine the duration of its potential effects. Methods: A 56-year-old woman diagnosed with frontotemporal dementia (FTD) and svPPA participated in this longitudinal single-subject design. A total of four 2-week stimulation phases were conducted over 14 months, each of which began depending on the participant’s language performance. Follow-up testing was conducted approximately 2 weeks and 4 weeks after the stimulation period. The authors presented results showing a significant improvement in word retrieval after SFA and tDCS therapy. Two weeks after the end of each stimulation phase, approximately 80% of the trained words could be named correctly. For the untrained words, also significantly more words were correctly named at follow-ups compared to the baseline. Furthermore, the Boston Naming Test (BNT) demonstrated a significant improvement in naming performance, showing that phonological cues facilitated word retrieval more effectively than semantic cues. The authors conclude that the combination of SFA and tDCS was able to counteract the expected language deterioration of a participant with svPPA. This effect increased until approximately 2 weeks after each intervention. Additionally, the impact on untrained words was also demonstrated.

## 3. Conclusions

This Special Issue included thirteen articles that reflected the new aspects in the fields of language, communication, and cognitive rehabilitation. As was presented before, the five (5) original articles focus on various conditions and disorders such as dysarthria, high-functioning autism, developmental language disorder, and cognitive decline. These studies discussed traditional methodologies and new technologies (large language models, brain stimulation, computer-based interventions). As indicated by the conclusions of all the studies, they provided vital and crucial insights for communication assessment and rehabilitation in individuals with these difficulties.

In addition, the other review studies (literature, scoping, and systematic reviews) presented data from the past two decades across various disorders like Rett syndrome, Aphasia, Locked-in syndrome, and Alzheimer’s disease. A general conclusion of the majority of these studies is that it underlined the need for personalized assessment and treatment with an interdisciplinary approach. Finally, this Special Issue has delivered a message that communication remains a fundamental element of human interaction, which can have a significant impact on the quality of life. We hope that this Special Issue will be a valuable resource for professionals, clinicians, academics, and researchers, enabling them to improve outcomes for individuals facing communication and language difficulties.
